# Structural insights into chondroitin sulphate A binding Duffy-binding-like domains from *Plasmodium falciparum*: implications for intervention strategies against placental malaria

**DOI:** 10.1186/1475-2875-8-67

**Published:** 2009-04-17

**Authors:** Jasmita Gill, Chetan E Chitnis, Amit Sharma

**Affiliations:** 1 Structural and Computational Biology Group, International Centre for Genetic Engineering and Biotechnology (ICGEB), Aruna Asaf Ali Road, New Delhi, 110067, India; 2 Malaria Research Group, International Centre for Genetic Engineering and Biotechnology (ICGEB), Aruna Asaf Ali Road, New Delhi, 110067, India

## Abstract

**Background:**

Placental malaria is typified by selective clustering of *Plasmodium falciparum *in the intervillous blood spaces of the placenta. Sequestration of malaria parasite in the human placenta is mediated by interactions between chondroitin sulphate A (CSA) on the syncytiotrophoblasts and proteins expressed on the surface of infected human erythrocytes. *Plasmodium falciparum *Erythrocyte Membrane Protein 1 (PfEMP1) encoded by the *var2CSA *gene is believed to be the main parasite ligand for CSA-mediated placental binding.

**Methods:**

Extensive sequence and structure comparisons of the various CSA-binding and non-binding DBL domains from the *var2CSA *gene from A4 and 3D7 strains of *P. falciparum *were performed. Three-dimensional structural models of various DBL domains were built and analysed with a view to assessing conservation of CSA interaction sites across various DBL domains.

**Results:**

Each of the six DBL domains from *var2CSA *are likely to retain the disulfide linkages evident from previously published DBL domain crystal structures. The number of disulfide linkages between the various DBL domains analysed varies from three to seven, of which two are conserved across all DBL domains. The conserved disulfide linkages are distributed within the respective three sub-domains and only one linkage is shared by sub-domains I and II. Major differences between CSA-binding DBL domains are in the loop regions, which tie the alpha helices together, and in variable length terminal extensions. Intriguingly, a crucial loop from A4 DBL 3X which provides the important Gly and Lys residues that chelate the bound sulphate is missing or significantly altered in all other DBL domains that interact with CSA. Further analysis of the proposed sulphate and predicted CSA-binding site indicates either none or very low level of conservation among the critical interacting residues.

**Conclusion:**

Structural comparisons of the three-dimensional structures of CSA-binding DBL domains indicates that the proposed CSA interaction site on A4 DBL 3X is unlikely to be conserved across the other CSA-binding DBL domains from *var2CSA*. Therefore, the 4 CSA-binding DBL domains encoded by *var2CSA *are unlikely to have common architectures to their CSA recognition sites. These structural insights have clear implications in using CSA-binding DBL domains for vaccines against placental malaria as it is proposed that the various CSA-binding DBL domains on *var2CSA *will recognize their CSA ligands differently.

## Background

It has long been observed that there is an increase in the severity of malaria during pregnancy, resulting in such negative outcomes as maternal anaemia and reduction in birth weight, which in turn lead to increase in maternal and infant mortality [1]. Adverse outcomes of pregnancy-associated malaria (PAM) include low birth weight neonates, foetal loss, increased perinatal and maternal mortality, maternal anaemia and the risk of hypertension in first-time pregnant mothers [2].

Pregnancy-associated malaria is coupled with massive accumulation of parasitized erythrocytes (PE) and monocytes in the placental intervillous blood spaces. The basis for the amassing of parasitized erythrocytes (PEs) in the placenta was unknown until it was shown that PEs from placenta primarily bind to chondroitin sulfate A (CSA) [3]. Significantly, after one or two pregnancies, antibodies that recognize placental PEs from different geographic regions develop and correlate with protection against PAM [3,4]. Antibodies against CSA-binding parasites that develop in multigravidae in endemic areas also block CSA-binding of placental isolates from different parts of the world, demonstrating the development of strain transcending antibodies to the *Plasmodium falciparum *ligands that mediate adhesion to CSA [5].

The *var2CSA *gene, which is a member of the *P. falciparum *Erythrocyte Membrane Protein 1 (PfEMP1) family, may have an important role in PAM disease and immunity. This gene encodes a large protein with an estimated molecular weight of 350 kDa, and can be divided into six Duffy-binding-like domains (DBL 1–6) based on several conserved cysteines. The DBL domains are cysteine-rich modules which recognize diverse host cell-surface receptors during pathogenesis. The gene *var2CSA *is found in all parasite isolates and is transcriptionally up-regulated in both placental isolates and laboratory parasites selected to bind CSA [6-9]. Significantly, *var2CSA *knock-out parasites revealed that no other parasite ligand can promote adhesion to CSA [6]. Furthermore, the *var2CSA *encoded protein contains multiple CSA-binding DBL domains and is the target of maternal antibodies, making it the leading candidate for malaria vaccine in pregnancy. [7-9].

In the present study, three-dimensional structures of all CSA-binding and non-binding DBL domains from A4 and 3D7 strains of *P. falciparum *were modelled and analysed by predicting the disulfide linkages using the crystal structures of A4 DBL 3X domain , *Plasmodium knowlesi *DBL domain Pkα-DBL and *P. falciparum *EBA-175 DBL domains F1 and F2 [10-13]. An overall comparison based upon the superposition of these domains shows that secondary structure elements are conserved and major differences between the various domains lie mainly within flexible loop regions and/or N/C termini. The number of disulfide linkages between various DBL domains analysed varies from three to seven, of which two are conserved across all domains. Intriguingly, the surface charge properties of each of these analysed DBL domains clearly indicate that there are no conserved patches of either positive or negative charge density. Further, none of these DBL domains harbour exposed hydrophobic patch of reasonable volume. A comparison of the proposed CSA-binding site (based upon the sulphate ion bound in A4 DBL 3X crystal structures [10,11]) shows that this binding site is unlikely to be conserved among different CSA-binding domains. The structural analysis and structure-based insights presented here have widespread implications in using CSA-binding DBL domains for vaccines against placental malaria.

## Methods

### Sequence alignments and structural modelling of *var2CSA *gene DBL domains

All sequence alignments were done using ClustalW2 online program. Structures of the DBL domains from A4 and 3D7 *var2CSA *were modelled using HHpred server with default settings [14]. The HHpred method is based on comparisons and alignments of hidden Markov models (HMMs), which include gaps and insertion probabilities [14]. The *var2CSA *DBL domains were modelled for 3D7 (PFL0030c, *Plasmodium *genomic database 'PlasmoDB') and for A4 strains (accession code AY372123). The PFL0030c sequence was split into separate domains (DBL 1X aa 46–343, DBL 2X aa 535–921, DBL 3X aa 1209–1559, DBL 4ε aa 1560–1982, DBL 5ε aa 1983–2079 and DBL 6ε aa 2323–2628). The A4 accession code AY372123 sequence was also split into separate domains (DBL 1X aa 46–344, DBL 2X aa 536–930, DBL 4ε aa 1578–1988, DBL 5ε aa 1989–2276 and DBL 6ε aa 2320–2631). All HMM databases available in web-server were used for template structure search, including the Protein Data Bank. Multi-template alignments proposed by the HHpred method were used to generate 3D models by using HHpred server toolkit protocol for MODELLER [15]. Structure superpositions were done using MatchMaker in Chimera [16]. All structural visualizations were produced using Chimera.

## Results and discussion

### Sequence alignments of *var2CSA *DBL domains

**Figure 1:**
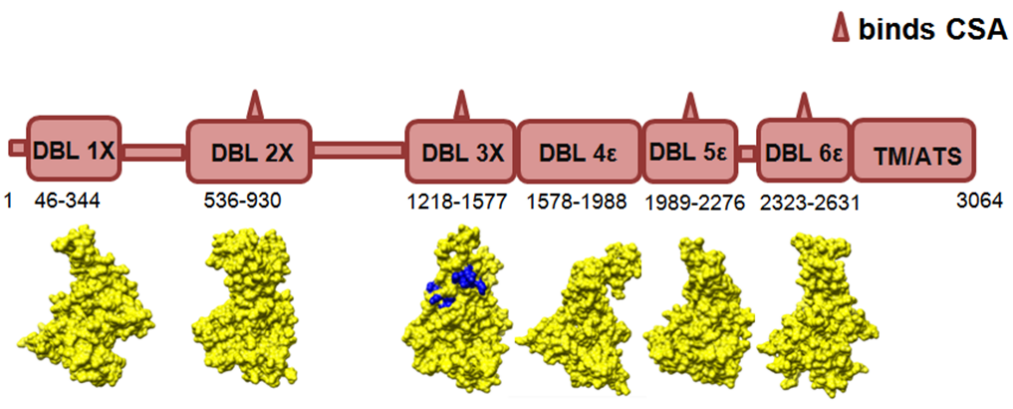
**Domain diagram of A4 *var2CSA *gene**. Distribution of residues that form various DBL domains is shown. Modelled structures for the corresponding domains are given below each domain box. All models are shown in surface representation and coloured yellow. The sulphate-binding residues on A4 DBL 3X crystal structure are coloured blue (PDB codes: 3BQK and 3CPZ).

The crystal structures of DBL 3X domain from A4 strain of *P. falciparum *have been recently determined with a single bound sulphate ion [10,11]. The protein sequences of all *var2CSA *encoded DBL domains from the A4 and 3D7 strains of *P. falciparum *(2X, 3X, 5ε and 6ε) that bind CSA and also two remaining non CSA-binding domains (1X and 4ε) were aligned with the A4 DBL 3X crystal structure sequence. Since all conserved cysteines in DBL domains where structures have been determined to date [10-13] make conserved disulfide linkages with other cysteines, this strongly suggests that DBL domains are constructed around conserved structural features like these S-S linkages. Therefore, in the sequence analysis of CSA-binding and non-binding DBL domains (with reference to the crystal structure of A4 DBL 3X), this structural property of DBL domains was utilized for generating sequence alignments and for defining the domain boundaries. After aligning the sequences, disulphide linkages in the structure of A4 DBL 3X were compared with sequences of 3D7 DBL 3X domain (Additional file 1) and with other 3D7 *var2CSA *DBL domains. Subsequently, predictions were made for the disulfide linkages of all DBL domains from A4 and 3D7. Based upon these predictions, final sequence boundaries for DBL domains were defined (Additional file 2). These sequence boundaries were then utilized for homology modelling of all *var2CSA *domains that bind CSA using the recently determined crystal structures of A4 DBL 3X and previously determined structures of *P. knowlesi *DBL domain Pkα-DBL and *P. falciparum *EBA-175 DBL domains F1 and F2 [10-13]. Sequence boundaries for the non CSA-binding DBL 1X and 4ε domains from 3D7 and A4 *var2CSA *respectively were also defined using a similar procedure. For the DBL 4ε domain, sequence boundary was defined as the entire sequence between DBL 3X and 5ε domains in *var2CSA *(Figure 1). These sequence boundaries were used for structural modelling of the CSA-binding and non-binding DBL domains (Figure 1).

### Structural modelling of the *var2CSA *DBL domains

Overall sequence identity between CSA-binding DBL domains of different sub-types from A4 *var2CSA *and 3D7 *var2CSA *is low (ranges from 13 to 28%) (Table 1). In contrast, the sequence identity between CSA-binding DBL domains of same sub-type is high (from 62 to 87%) (Table 1). After sequence boundaries had been defined for all CSA-binding and non-binding domains (Figure 1, Additional file 2), 3D structures of these 3D7 and A4 DBL domains were modelled. The template search in HHpred listed the recently determined structure of *P. falciparum *A4 DBL 3X [10], *P. knowlesi *DBL domain Pkα-DBL [12] and *P. falciparum *EBA-175 DBL domains F1 and F2 [13]. Utilizing all these templates, multi-template models of a total of seven CSA-binding domains (3D7 *var2CSA *DBL 2X, 3X, 5ε and 6ε domains; A4 *var2CSA *DBL 2X, 5ε and 6ε domains) and two non-binding domains (3D7 *var2CSA *DBL 1X and A4 var2CSA DBL 4ε) were generated. The structures for DBL domains 3D7 DBL 1X and 3X; A4 and 3D7 DBL 5ε and A4 and 3D7 DBL 6ε were modelled successfully in the first run. However, for 3D7 and A4 DBL 2X domains, the program inserted a loop region in the middle of a crucial and conserved α-helix 2 in sub-domain II – a result which is unlikely. Hence, to successfully generate correct models for 2X domain, the input sequence length for the program was altered while keeping conserved disulfides intact in this altered 2X sequence length (Figure 1). Models for non-binding DBL 1X and 4ε domains were used as controls for comparative structural analysis of CSA-binding DBL domains. Since the entire sequence length between DBL 3X and 5ε domains was used to model DBL 4ε domain, it was observed that the N- and C- terminal regions of DBL 4ε are longer and flexible when compared to other DBL domains.

**Table 1:** Sequence identity within the *var2CSA *DBL domains from 3D7 and A4 (in percentage)

	**CSA-binding domains**								**Non binders**	
	**3D7**				**A4**				**3D7**	**A4**

	**2X**	**3X**	**5ε**	**6ε**	**2X**	**3X**	**5ε**	**6ε**	**1X**	**4ε**

**3D7 2X**	-									

**3D7 3X**	23	-								

**3D7 5ε**	21	17	-							

**3D7 6ε**	21	18	28	-						

**A4 2X**	80	19	23	21	-					

**A4 3X**	24	85	17	16	18	-				

**A4 5ε**	24	18	87	27	27	17	-			

**A4 6ε**	22	19	25	62	18	16	24	-		

**3D7 1X**	23	20	18	16	26	20	23	18	-	

**A4 4ε**	17	16	21	26	13	16	24	23	19	-

**Figure 2:**
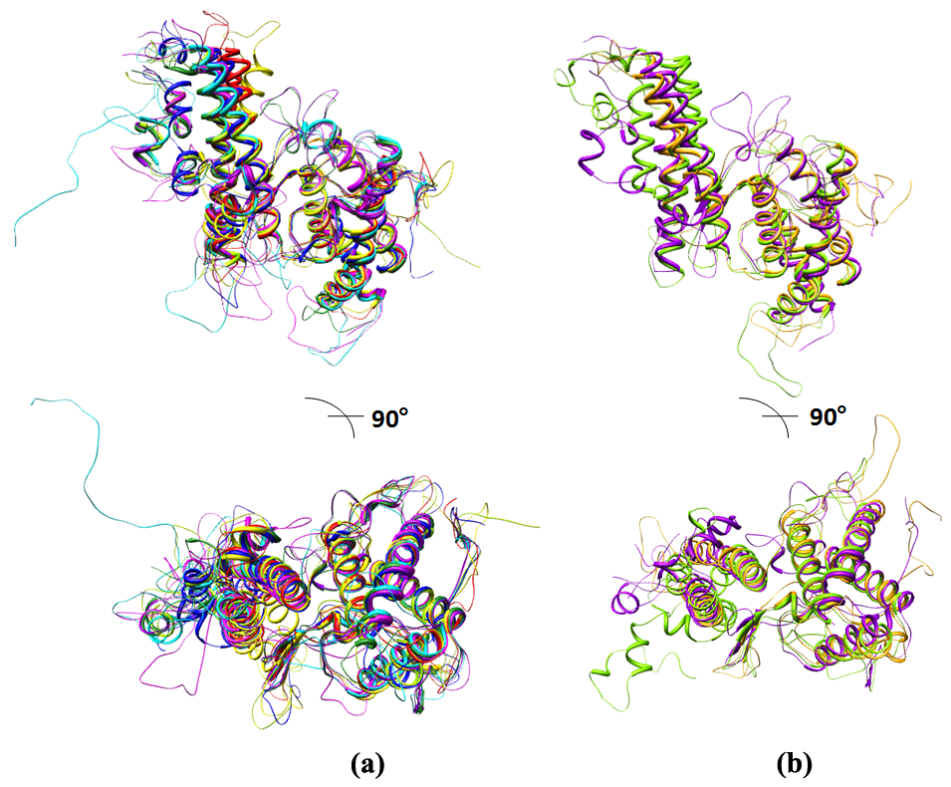
**Analysis/comparison of CSA-binding and non-binding DBL domains with A4 DBL 3X (PDB code: **2BQK**).** (a) Superposition of CSA-binding A4 DBL 2X, 5ε, 6ε domains and 3D7 DBL 2X, 3X, 5ε and 6ε domains onto A4 DBL 3X structure. (b) Superposition of non-CSA-binding DBL domains 3D7 DBL 1X and A4 DBL 4ε on to A4 DBL 3X structure.

Structural superpositions of the seven domains that bind CSA and the two non-binding domains were performed on to the A4 DBL 3X crystal structure (10; PDB code: 3BQK) (Figure 2a, b). A structure-based sequence alignment was subsequently generated keeping the A4 DBL 3X crystal secondary structure as a control (Additional file 4). The disulfide linkage predictions made earlier on the basis of sequence alignments with the A4 DBL 3X crystal structure were confirmed upon generation of these models (Table 2).

**Table 2:** Disulfide linkages in CSA-binding DBL domains

	Strain and DBL domain	Disulfide linkages based on A4 DBL 3X	Additional predicted disulfides	Likely free cystein(s)
	A4 3X crystal structure	8	-	None

1	3D7 3X	7	-	2 (C1212, C1558)

2	3D7 2X	4	C793 and C920	10 (C540, C543, C592, C732, C733, C786, C797, C815, C855, C901)

3	A4 2X	3	C795 and C910 C817 and C877	9 (C544, C547, C599, C617, C734, C788, C799, C807, C929)

4	3D7 5ε	3	C2004 and C2041	2 (C1995, C2165)

5	A4 5ε	3	C2010 and C2047	2 (C2001, C2171)

6	3D7 6ε	3	-	7 (C2334, C2385, C2480, C2539, C2602, C2604, C2625)

7	A4 6ε	3	-	8 (C2331, C2383, C2478, C2493, C2535, C2597, C2599, C2628)

### Structural comparisons between CSA-binding DBL domains

All the modelled domain structures contain sub-domains I, II and III similar to the crystal structures of *P. falciparum *A4 DBL 3X, *P. falciparum *EBA-175 DBL domains F1 and F2 and *P. knowlesi *DBL domain Pkα-DBL [10-13] as has been shown earlier [17]. Further, the overall alpha-helical content in sub-domains II and III is conserved in all modelled DBL domains. Structural differences lie mostly in loop regions between helices and/or in the N- and/or C-termini which are flexible and could assume variable orientations. Sub-domain I contains an N-terminal region of variable residue length which is highly flexible. In sub-domain II, the loop regions/random coils between α-helices 1 and 2 and α-helices 3 and 4 are longer in the DBL 3X and 2X domains respectively when compared to the other domains. The C-terminal region is also comparatively longer for A4 and 3D7 DBL 2X domains mainly because the A4 DBL 2X domain has larger number of residues in A4 (395 aa). These extra residues in DBL 2X domains are mostly inserted in flexible loops regions between the helices in sub-domains II and III and/or in C-terminal regions. The DBL 4ε domain consists of 411 residues and possesses comparatively longer termini that are probably flexible in nature. The regions which have not been modelled for *var2CSA *domains are the non-DBL domain stretches from A4 at the start (aa 1–45), between DBL 1X and 2X (aa 345–535), between 2X and 3X (aa 931–1217), between 5ε and 6ε domains (aa 2277–2322) and in the TM/ATS region (aa 2632–3064) (Figure 1).

The DBL domains of A4 and 3D7 *var2CSA *have three to seven conserved disulfide linkages (Table 2). Upon analysing distribution of these disulfide linkages in various sub-domains, it was noted that sub-domains I and II each contain two and one conserved linkages respectively (based on the A4 DBL 3X crystal structure) while sub-domain III contains three conserved and three predicted linkages (based on structural modelling) (Additional file 4, Figure 3). There is only one conserved inter-domain disulfide bond which links sub-domains I and II, whereas all other disulfide bonds are intra sub-domain linkages (Additional file 4, Figure 3, 4) [12,13]. In sub-domain I, both the conserved disulfide linkages occur in loop regions. In sub-domain II, the conserved disulfide links α-helix 2 and a loop region. Overall, there are two disulfide linkages that are conserved in all the CSA-binding DBL domains and these lie in sub-domain III (Additional file 4, Figure 3).

**Figure 3:**
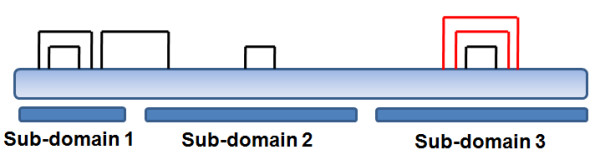
**Distribution of disulfide linkages amongst the 3 sub-domains**. Sub-domain I, II and III each contain 2, 1 and 3 conserved disulfide linkages respectively (shown as lines). The 2 disulfides in sub-domain III (coloured red) are conserved in all CSA-binding DBL domains.

**Figure 4:**
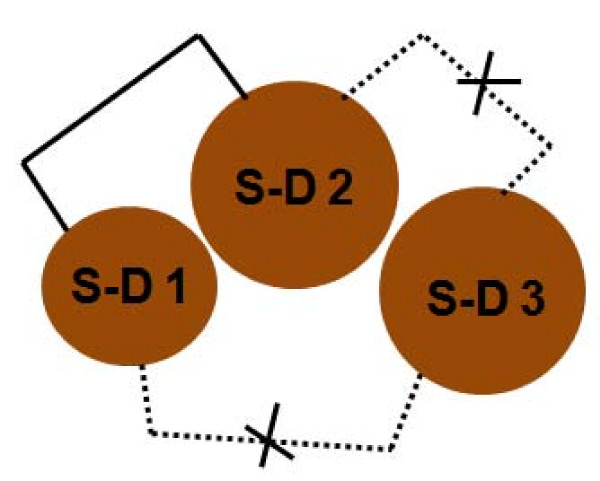
**Inter and intra-domain disulfide linkages in DBL domains**. For all DBL domains, only sub-domain I and II share 1 inter-domain disulfide linkage whereas there are no inter-domain linkages between sub-domains I and III and sub-domains II and III.

### A consideration of the sulphate binding site from A4 DBL 3X crystal structures

**Figure 5:**
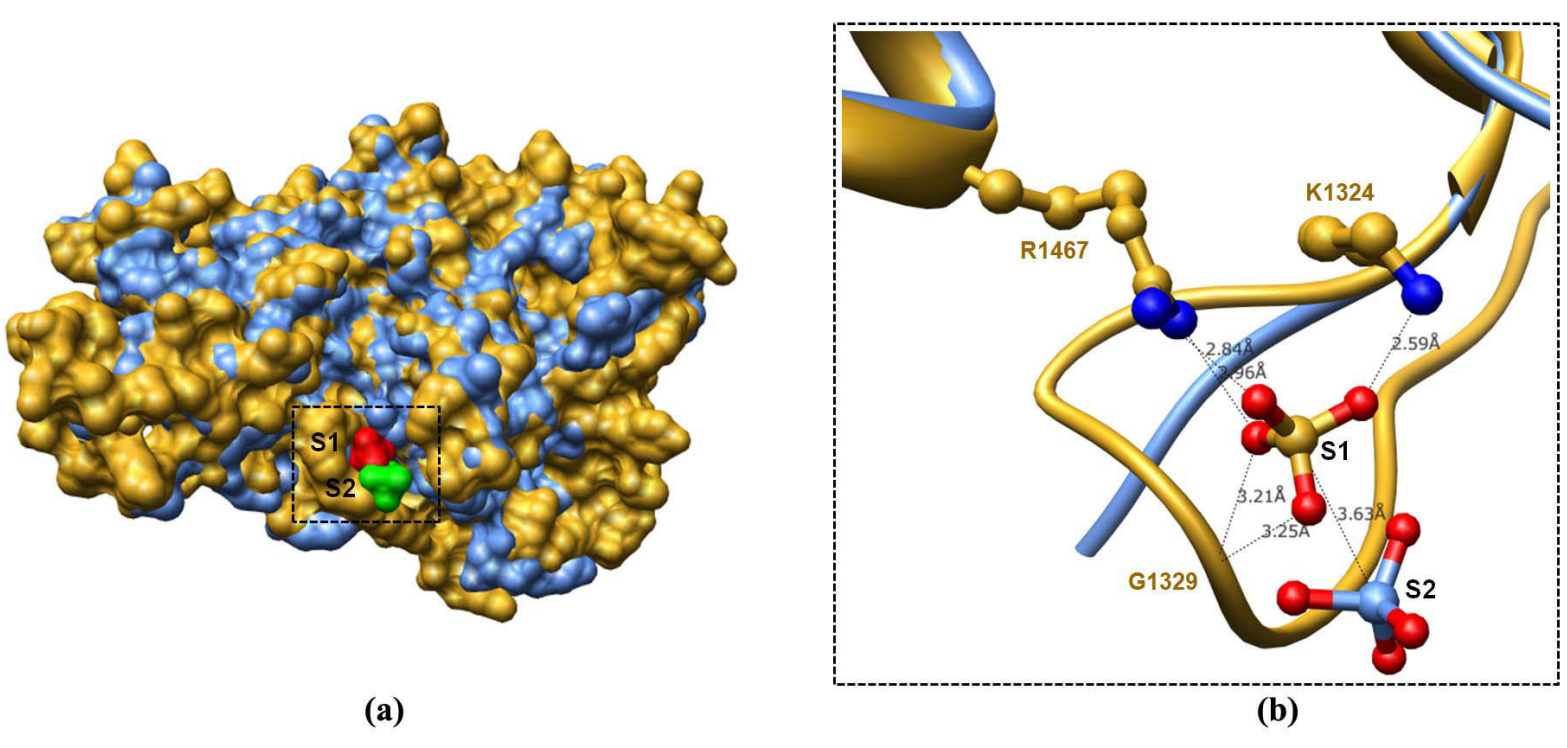
**Superposition of A4 DBL 3X crystal structures (PDB codes: **2BQK** and **3CPZ**)**. (a) Superposition shown in surface representation. The A4 DBL 3X crystal structures are coloured brown (PDB code: 3BQK) and blue (PDB code: 3CPZ. (b) Close view of the sulphate-binding site. The sulphate ions bound to the structures are labelled S1 (green; PDB code: 2BQK) and S2 (red; PDB code: 3CPZ) respectively. S1 makes contacts with residues Lys1324, Gly1329 and Arg1467. The sulphate ion S2 however does not make protein residue contacts and the distance of its sulphur atom from the sulphur atom of S1 is ~3.6Å.

**Figure 6:**
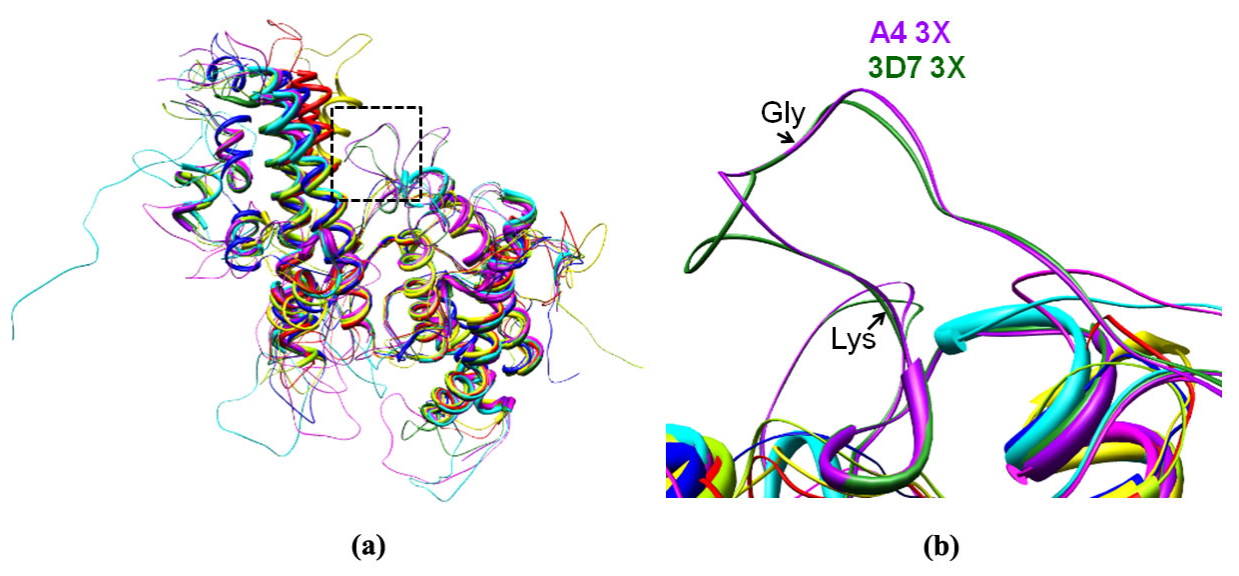
**Comparison of sulphate binding site in all the CSA-binding *var2CSA *DBL domains**. (a) Superposition of all CSA-binding DBL domains onto the A4 DBL 3X crystal structure (PDB code: 3BQK). The sulphate-binding site is highlighted. (b) Close view of the sulphate-binding site. The loop region bearing the important Gly and Lys residues is magnified. This loop is either absent or is in a different orientation in all CSA-binding domains except A4 (magenta) and 3D7 (green) DBL 3X.

In the crystal structure of A4 DBL 3X (10; PDB code: 3BQK; Figure 5a, b), a single sulphate ion is bound in a pocket located between a loop at a turn between α-helices 1 and 2 of sub-domain II and makes contact with a residue from α-helix 1 of sub-domain III. This proposed binding site contains two charged basic residues (Lys1324 and Arg1467) and a Gly1329 which together interact with the sulphate ion. Lys1324 and Gly1329 lie in the loop whereas Arg1467 lies towards the end of α-helix 1 of sub-domain III. Lys1324 forms hydrogen bonds with Arg 1467 and enables Gly1329 to interact with the sulfate ion [10]. These residues from the crystal structure were compared with the corresponding residues from the other CSA-binding DBL domains (Table 3). The α-helix which contains residue Arg1467 shows poor sequence conservation amongst CSA-binding domains except in A4 DBL 6ε. Replacement of an Arg/Lys residue by a Gln may retain the ability to form a hydrogen bond with the sulphate ion, but without the electrostatic component in the binding interface. Further, Arg1467 is replaced by Gln in 3D7 DBL 3X, which displays high sequence similarity with the A4 DBL 3X. Interestingly, this Arg1467 is replaced by Glu residue in 3D7 DBL 2X, 5ε, 6ε and A4 DBL 5ε – conferring an opposite charge to this important residue. Furthermore, in 3D7 DBL 2X and 1X domains there is replacement by Ala and Asn residues respectively (Table 3). The other two residues involved in binding to sulphate, Lys1324 and Gly1329, are present in the loop region between the α-helices 1 and 2 of sub-domain II (Figure 6a, b). In 3D7 DBL 3X domain, the corresponding Arg and Gly residues are present thus indicating that the mode of sulphate binding is, not surprisingly (just a difference in parasite strains), likely to be conserved in the DBL 3X domains, as has been earlier suggested [10]. However for the other domains, this region is highly flexible and the corresponding residues for Lys and Gly are absent in the sequence/structural alignment of these domains (Figure 6a, b).

**Table 3:** Comparison of residues implicated in sulphate binding

	**CSA-binding DBL domains**							**Non-binders**	
**A4 3X**	**3D7 3X**	**2X**		**5ε**		**6ε**		**A4 4ε**	**3D7 1X**

		A4	3D7	A4	3D7	A4	3D7		

Sulfate binding residues									

K 1324*	**R**	**-**	-	-	-	-	-	**H**	-

G 1329*	**G**	**-**	-	**K**	-	-	-	**-**	-

R1467*	**Q**	**A**	*E*	*E*	*E*	**K**	*E*	**K**	**N**

K1504 ^#^	**K**	*E*	**K**	*E*	*E*	**A**	**A**	**A**	**K**

Residues within 5Å of modelled CSA									

K1327^#^	**M**	-	**K**	**-**	**K**	-	-	-	

R1503^#^	**R**	*D*	**N**	**S**	**S**	**H**	*E*	**K**	*D*

K1507^#^	*E*	**K**	**V**	**K**	**K**	**N**	**N**	**N**	**K**

K1510^#^	**K**	**K**	**K**	*E*	*E*	**N**	**N**	**N**	*E*

• [10,11].

• Conserved residues are in bold, acidic residues are in italics and underlined and other residues are bold and underlined.

Another crystal structure of the A4 DBL 3X with a single sulfate ion bound has been determined recently [[11]; PDB code: 3CPZ]. However, in this second structure, the residue environment is absent for the sulphate ion and upon the superposition of the 2 crystal structures of A4 DBL 3X, the position of the sulphur atom differs by a distance of ~3.6 Å (Figure 5a, b). Further, one additional residue – Lys1540 – is implicated in making contact with the sulphate ion. Apart from this residue, four other residues (Lys1327, Arg1503, Lys1507 and Lys 1510) have been highlighted which all are at a distance of ~5Å from the modelled CSA in the crystal structure [11]. The corresponding residues implicated in binding to modelled CSA [11] were further analysed in all CSA-binding domains (Table 3). The corresponding residues for the non-binding DBL 1X and 4ε domains were also highlighted. From this comparison, it is again evident that the site where the sulphate ion binds [11] is not conserved amongst the CSA-binding DBL domains (Table 3). The residue Lys1504 (though it does not make any contact with the sulphate ion; PDB code: 3CPZ) has a corresponding Lys in 3D7 DBL 3X, 2X and the non-binding DBL 1X domains (Table 3). However, it is replaced by Glu or Ala in other CSA-binding DBL domains. The other four residues implicated in CSA binding (at interacting distance of ~5Å distance) are also not conserved in these domains (Table 3). These sulphate binding residues were also compared with the corresponding ones in DBL 3γ domain of v*ar1CSA *which binds CSA [9]. The proposed binding site is, once again, not conserved in DBL 3γ although all the three disulfide linkages predicted for DBL 3γ domain are in accordance with the A4 DBL 3X crystal structure.

**Figure 7:**
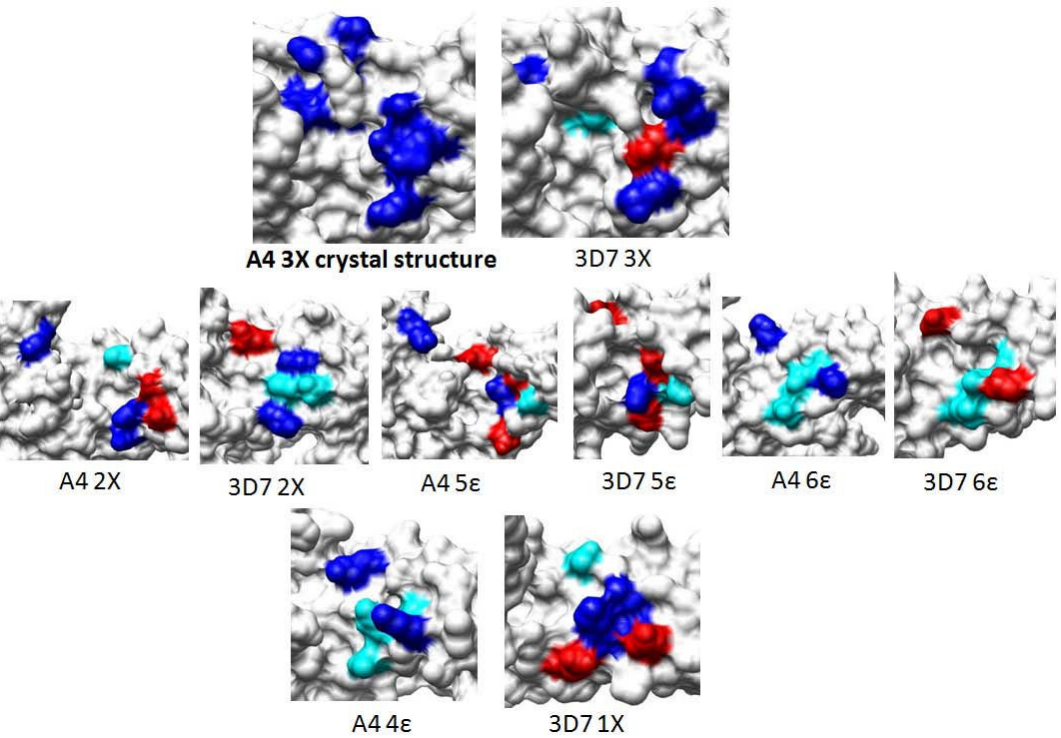
**Comparison of sulphate-binding residues in *var2CSA* DBL domains.** A4 DBL 3X crystal structure is used as control and all residues involved in binding to sulphate ion are coloured blue (PDB codes: 3BQK and 3CPZ). Residues which are conserved in other domains are also colored blue. Residues which are replaced with acidic amino acids are coloured red. Residues replaced by other residue types are coloured cyan.

Thus, comparison of the sulphate binding residues (from the two crystal structures) in these DBL domains shows that the sulphate binding site is only very partially conserved in the seven CSA-binding DBL domains from *var2CSA *(Figure 6, 7; Table 3) and in one DBL domain from *var1CSA*. Interestingly, few of these residues are conserved in DBL 1X and 4ε domains which do not bind CSA (Figure 7; Table 3). This lack of conservation in the sulphate binding site is also evident from the low sequence identity shared by these DBL domains (Table 1). Thus, it is unclear whether the crystal structure-based sulphate ion binding sites will interact with sulphate from CSA in a similar manner in other DBL domains. Since the proposed sites only bind a single sulphate ion in current crystal structures, it may be too early to speculate whether this is strictly the region which can be defined as the CSA recognition site in other CSA-binding DBL domains.

Conservation in the CSA-binding sites within all four DBL domains of a given *var2CSA *gene is intuitively, and from the presented data evidentially, unfeasible and unlikely. Instead, it is likely that CSA-binding DBL domains have evolved to differ in their regions which recognize CSA – a notable feature of significance if the parasite needs to evade immune responses efficiently. As an extension of this argument, it is proposed that it may be unlikely that a common inhibitor/drug will bind to the CSA-recognition pocket in all CSA-recognizing DBL domains. In summary, it is plausible that CSA makes different (potentially non-overlapping) binding footprints on various CSA-binding DBL domains.

### Analysis of conserved regions on CSA-binding DBL domains

**Figure 8:**
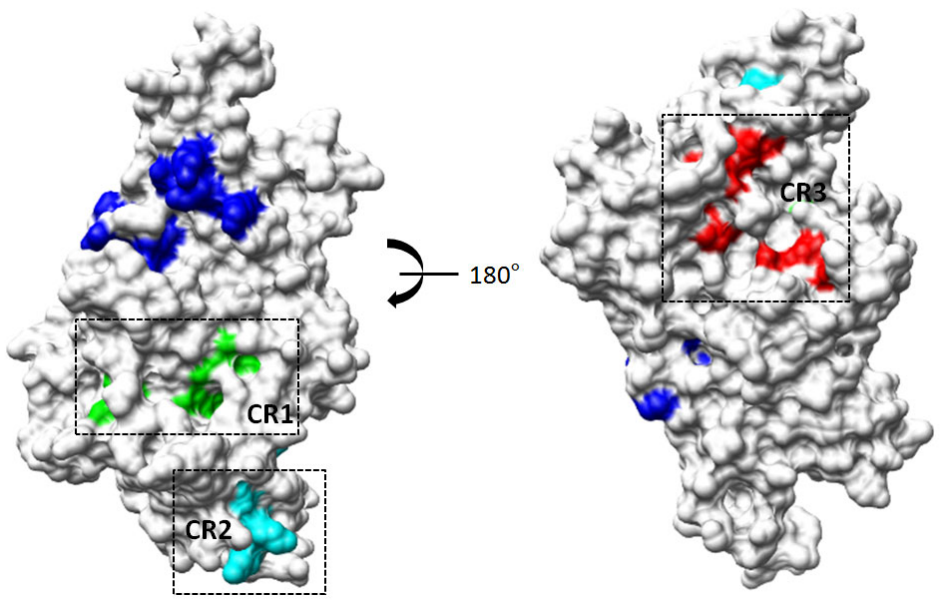
**Mapping of identical/conserved surface residues on A4 DBL 3X that are invariant and exposed in all CSA-binding DBL domains**. Residues constituting the three conserved regions identified CR1, CR2 and CR3 are coloured green, cyan and red respectively.

To investigate if there are other regions on these DBL domains which have identical/conserved and exposed residues, the surfaces of DBL domains were inspected (keeping in view sequence and structural information from crystal structure of A4 DBL 3X [10]). These residues were then highlighted amongst the DBL domains based on the described criterion (Additional file 3, Figure 8). Based upon identification of identical/conserved surface residues, three new regions are proposed on A4 DBL 3X which are conserved between all CSA-binding DBL domains (Figure 8). Two of these regions (named conserved region – CR1 and CR2) are on the same face as the sulphate binding site (proposed CSA-binding site) and a third region (named CR3) is on the opposite face. The cavity formed by CR1 consists of conserved residues Ile1265, Gln1270, Leu1272, Leu1310, Asp1353, Met1357 and Gln1449 – which lie mostly in sub-domain I while few lie in α-helices 1 and 2 of sub-domain II. The region constituting CR2, which lies on the same face as the sulphate binding site, has more hydrophilic residues when compared to CR1, including exposed lysine residues Lys1378 and Lys1382. The other residues constituting CR2 are Ile1358, Gly1360, Val1363 and Trp1404. These residues lie mostly between α-helices 2 and 3 of sub-domain II. The third conserved region CR3, which is on the opposite face of the sulphate binding site, and has a line up of residues such as Phe1351, Trp1405, Trp1413, Pro1441, Val1451, Lys1455 and Tyr1526. The residues constituting this region are scattered in α-helices 1, 2 and 4 of sub-domain II and in α-helices 1 and 2 of sub-domain III. All these three conserved regions lie only in sub-domain I and II except for a few residues of CR3 which emanate from sub-domain III. It is interesting to note the presence of these conserved CR1–CR3 regions on different CSA binding DBL domains (Figure 8). The exact relevance of these three conserved regions is not yet clear but they may play a structural role in these DBL domains. If so, these sites may be potential new targets for development of small molecules that target the overall functioning of DBL domains.

In an earlier study, antibody binding regions have been predicted on DBL domains by identifying linear epitopes and mapping them onto the modelled DBL structures [17]. For the CSA-binding DBL 2X, 3X, 5ε and 6ε domains, the epitope regions lie exclusively in sub-domain I and II [17]. Interestingly, for DBL 4ε, which does not bind CSA, the epitope region also covers the sub-domain III. The proposed CSA-binding site lies mostly in sub-domain III [10,11]. The identified regions CR1 and CR2 are only in sub-domain I and II and only a few residues from the CR3 lie in sub-domain III. Also, few residues of conserved regions CR1 and CR2 that lie exclusively in sub-domain I and II are in proximity and/or overlap with this proposed antibody binding region [17]. Whether such conserved B cell epitopes can serve as targets for strain-transcending antibodies directed against these CSA-binding domains remains to be determined. Whether any of these conserved surfaces on CSA-binding DBL domains can serve as targets for B cell epitopes is also unknown as yet.

## Conclusion

In the present study, three-dimensional structures of CSA-binding DBL domains from *var2CSA *– the main parasite ligand for human placental CSA – were predicted and analysed. The analysed DBL domains have a variable number of disulfide linkages out of which three to seven are conserved in accordance with the A4 DBL 3X crystal structure. The described detailed sequence and structural analysis suggests that the CSA-binding DBL domains from *var2CSA *and *var1CSA *are unlikely to retain similar/identical CSA recognition surfaces. This crucial observation seems consistent with the need for *P. falciparum *to constantly evade immune responses against its CSA recognizing DBL domains. Maintenance of identical CSA interacting sites on the four DBL domains from *var2CSA *will provide an easier immune target than a scenario where the four DBL domains use different and variable surfaces for CSA chelation. It is more evident than before that a complete description of CSA-binding footprints on CSA-binding DBL domains will be of tremendous value as a platform to further guide efforts at drug/vaccine development against placental malaria.

## Competing interests

The authors declare that they have no competing interests.

## Authors' contributions

JG and AS designed the study. JG conducted all experiments and performed the analyses. JG wrote the manuscript in consultation with CC and AS.

## Supplementary Material

Additional file 1 **Sequence alignment of A4 DBL 3X (PDB code: **3BQK**) and 3D7 DBL 3X domains**. The data provided shows sequence alignment of A4 DBL 3X (PDB code: 3BQK) and 3D7 DBL 3X domains. The disulfide linkages of A4 DBL 3X structure are colored in pairs. Click here for file

Additional file 2 **Final sequence boundaries defined for the *var2CSA *CSA-binding DBL domains**. The data provided shows final sequence boundaries defined for all the CSA-binding DBL domains from A4 and 3D7 strains. Click here for file

Additional file 3 **Structure-based sequence alignment of CSA-binding DBL domains from A4 and 3D7 *var2CSA***. The data provided shows structure-based sequence alignment of CSA-binding DBL domains from A4 and 3D7 *var2CSA *highlighting the surface identical/conserved residues. Click here for file

Additional file 4 **Structure-based sequence alignment of CSA-binding DBL domains from A4 and 3D7 *var2CSA*.** The data provided shows structure-based sequence alignment of CSA-binding DBL domains from A4 and 3D7 *var2CSA *highlighting the conserved and predicted disulfide linkages in accordance with the A4 DBL 3X crystal structure. Click here for file
