# Supplementary material for: Structural insights into chondroitin sulphate A binding Duffy-binding-like domains from Plasmodium falciparum: implications for intervention strategies against placental malaria
Source: Malar J. 2009 Apr 17;8:67. doi: 10.1186/1475-2875-8-67 (PMC2676308; doi:10.1186/1475-2875-8-67)
Supplement: Additional file 1 — Sequence alignment of A4 DBL 3X (PDB code: 3BQK) and 3D7 DBL 3X domains. The data provided shows sequence alignment of A4 DBL 3X (PDB code: 3BQK) and 3D7 DBL 3X domains. The disulfide linkages of A4 DBL 3X structure are colored in pairs. [file 1475-2875-8-67-S1.pdf]

**Additional file 1:** Sequence alignment of A4 DBL 3X (PDB code: 3BQK) and 3D7 DBL 3X domains. The disulfide linkages of A4 DBL 3X structure are colored in pairs. The cysteins forming disulfide linkages in 3D7 DBL 3X are predicted and are colored cyan.

```
3D7-DBL3X  1209 SCDCSEPIYIRGCQPKIYDGKIFPGKGGEKQWICKDT------IIHGDTNGACIPPRTQN
A4-DBL3X   1218 SCDLNATNYIRGCQSKTYDGKIFPGKGGEKQWICKDT------IIHGDTNGACIPPRTQN
                         : .*  *  . *   .* .:*:** *.:      : :  :*   :*****.

3D7-DBL3X       LCVGELWDKRYGGRSNIKNDTKESLKQKIKNAIQKETELLYEYHDKGTAIISRNPMKGQK
A4-DBL3X        LCVGELWDKSYGGRSNIKNDTKELLKEKIKNAIHKETELLYEYHDTGTAIISKNDKKGQK
                **:    *:        *....: **:  .*:   :  ::  :*:       :*   .::

3D7-DBL3X       EKEEKNNDSNGLPKGFCHAVQRSFIDYKNMILGTSVNIYEYIGKLQEDIKKIIEKGTTKQ
A4-DBL3X        GK----NDPNGLPKGFCHAVQRSFIDYKNMILGTSVNIYEHIGKLQEDIKKIIEKGTPQQ
                 *    ** **  * :*:*:: ** ** ::* ***:   *:  .*: :::**: *   :

3D7-DBL3X       NGKT--VGSGAEN------VNAWWKGIEGEMWDAVRCAITKINKKQKKNGTFSID-ECGI
A4-DBL3X        KDKIGGVGSSTEN------VNAWWKGIEREMWDAVRCAITKINKK-NNNSIFNGD-ECGV
                  *   . ..:.        ::**:  :  :* *:: .    ...   :. .. . ..

3D7-DBL3X       FPPTGNDEDQSVSWFKEWSEQFCIERLQYEKNIRDACTNNG------------QGDKIQG
A4-DBL3X        SPPTGNDEDQSVSWFKEWGEQFCIERLRYEQNIREACTINGKNEKKCINSKSGQGDKIQG
                  ** :   * : :::** *:** :* .  : : : *.                ..: :

3D7-DBL3X       DCKRKCEEYKKYISEKKQEWDKQKTKYENKYVGK----------------------SAS
A4-DBL3X        ACKRKCEKYKKYISEKKQEWDKQKTKYENKYVGK----------------------SAS
                 **.*** ***:*.: *        :. ::                            .*

3D7-DBL3X       DLLKENYPECISANFDFIFNDNIEYKTYYP----------------------YGDYSSIC
A4-DBL3X        DLLKENYPECISANFDFIFNDNIEYKTYYP----------------------YGDYSSIC
                .  :    :*:.:::* :*:. *:     *                      *  *::
3D7-DBL3X       SCEQVK---------- 1559
A4-DBL3X        SCE------------- 1577
                .*:
```
