# Supplementary material for: Structural insights into chondroitin sulphate A binding Duffy-binding-like domains from Plasmodium falciparum: implications for intervention strategies against placental malaria
Source: Malar J. 2009 Apr 17;8:67. doi: 10.1186/1475-2875-8-67 (PMC2676308; doi:10.1186/1475-2875-8-67)
Supplement: Additional file 2 — Final sequence boundaries defined for the var2CSA CSA-binding DBL domains. The data provided shows final sequence boundaries defined for all the CSA-binding DBL domains from A4 and 3D7 strains. [file 1475-2875-8-67-S2.pdf]

Additional file 2: Final sequence boundaries defined for the *var2CSA* CSA-binding DBL domains from A4 and 3D7.

DBL domain	Protein sequence
**3D7 : PFL0030c, www.plasmodb.org**	
2X	535-921
3X	1209-1559
5ε	1983-2279
6ε	2323-2628
**A4: accession code AY372123**	
2X	536 - 930
5ε	1989-2276
6ε	2320-2631
