# Supplementary material for: Structural insights into chondroitin sulphate A binding Duffy-binding-like domains from Plasmodium falciparum: implications for intervention strategies against placental malaria
Source: Malar J. 2009 Apr 17;8:67. doi: 10.1186/1475-2875-8-67 (PMC2676308; doi:10.1186/1475-2875-8-67)
Supplement: Additional file 3 — Structure-based sequence alignment of CSA-binding DBL domains from A4 and 3D7 var2CSA. The data provided shows structure-based sequence alignment of CSA-binding DBL domains from A4 and 3D7 var2CSA highlighting the surface identical/conserved residues. [file 1475-2875-8-67-S3.pdf]

Additional file 3: Structure-based sequence alignment of CSA-binding DBL domains from A4 and 3D7 *var2CSA*. The disulfide linkages are colored in pairs. ‘S’ and ‘H’ denote sheet and helix region respectively for the A4 DBL 3X crystal structure (PDB code: 3BQK). The regions underlined in the N- and C- terminal regions could not be completely aligned. Residues underlined and in italics are disordered in A4 DBL 3X. Residues forming secondary structure are shown in bold. Surface identical/conserved residues constituting regions CR1, CR2 and CR3 are colored green, cyan and red respectively

```
                  ---------------------------------------SSS-------------
A4-DBL3X    1218      --SCDLNATNYIRGCQSKT-----YDGKIFPGKGGEKQWICKD-TIIHG-D---- 1259
3D7-DBL3X   1209      --SCDCSEPIYIRGCQPKI-----YDGKIFPGKGGEKQWICKD-TIIHG-D---- 1250
3D7-DBL2X   535    ASLTNCYKCDKCKSEQSKKNNK-----NWIWKKSSGKEGGLQ--KE----YA-N---- 576
A4-DBL2X    536    ASLTNGYKCDKCKSGTSRSKKK-------------------WIWKK-SSGNE-EGLQE 572
A4-DBL5ε    1989       DRCFDDQTKMKVCDLIADAIGCKDKTKLD----ELDEWNDMDLRGTYN-K---- 2033
3D7-DBL5ε   1983       DRCFDDKSKMKVCDLIGDAIGCKHKTKLDEL----DEWNDVDMRDPYN-K---- 2027
A4-DBL6ε    2320  HHEYDKGNDYICNKYKNIHDRMKK-----NNGNFVTDNFVKKSW---------E-I---- 2360
3D7-DBL6ε   2323      HHEYD-KGNDYI--C-NKY-----KNINVNMKKNNDDTW--TD-LVKNSSD---- 2361

                 ------SSS-HHHHH---HHH---HHH------------HHHHHHHHHHHHHHHHHHHHHH
A4-DBL3X    1260 --TN-GACIPPRTQNLCVGELWDKSYGGRSNIKNDT---KELLKEKIKNAIHKETELLYEY 1314
3D7-DBL3X   1251 --TN-GACIPPRTQNLCVGELWDKRYGGRSNIKNDT---KESLKQKIKNAIQKETELLYEY 1305
3D7-DBL2X   577  --TI-G--LPPRTQSLCLVVCLDEKGKKTQELKNIR-TNSELLKEWIIAAFHEGKNLKPS- 630
A4-DBL2X    573  EYAN-TIGLPPRTQSLYLGNLPKLENVCEDVKDINFDTKEKFLAGCLIVSFHEGKNLKKRY 632
A4-DBL5ε    2034 --HK-GVLIPPRRRQLCFSRIVR----GPANLRS----LNEFKEEILKGAQSEGKFLGNY- 2082
3D7-DBL5ε   2028 --YK-GVLIPPRRRQLCFSRIVR----GPANLRN----LKEFKEEILKGAQSEGKFLGNY- 2076
A4-DBL6ε    2361 --SN-GVLIPPRRKNLFLYIDPSK----ICEYKKD---PKLFKDFIYWSAFTEVERLKKA- 2410
3D7-DBL6ε   2362 --INKGVLLPPRRKNLFLKIDESD----ICKYKRD---PKLFKDFIYSSAISEVERLKKV- 2412

                 -------------------------------HHHHHHHHHHHHHHHHH----------HH
A4-DBL3X    1315 HDTGTAIISKNDKKGQKGK----NDPNGLPKGFCHAVQRSFIDYKNMILGTSV---NIYEH 1368
3D7-DBL3X   1306 HDKGTAIISRNPMKGQKEKEEKNNDSNGLPKGFCHAVQRSFIDYKNMILGTSV---NIYEY 1363
3D7-DBL2X   631  ----------HEK-----K----NDDNG--KKLCKALEYSFADYGDLIKGTSI---WDNEY 667
A4-DBL2X    633  PQNKN--------------------SGNKENLCKALEYSFADYGDLIKGTSI---WDNEY 669
A4-DBL5ε    2083 -------------YKEHKDK----------EKALEAMKNSFYDYEDIIKGTDM---LTNIE 2117
3D7-DBL5ε   2077 Y------NEDKDK-----------------EKALEAMKNSFYDYEYIIKGSDM---LTNIQ 2111
A4-DBL6ε    2411 Y-----------------------GGARAKVVHAMKYSFTDIGSIIKGDDMMEKNSSDK 2446
3D7-DBL6ε   2413 Y--GEA----------------------KTKVVHAMKYSFADIGSIIKGDDMMENNSSDK 2443

                 HHHHHHHHHHHHHHH---------------------HHHHHHHHHHHHHHHHHHHHHHHHHH
A4-DBL3X    1369 IGKLQEDIKKIIEKGTPQQKDK------IGGVGSSTENVNAWWKGIEREMWDAVRCAITKI 1422
3D7-DBL3X   1364 IGKLQEDIKKIIEKGTTKQNGK------T--VGSGAENVNAWWKGIEGEMWDAVRCAITKI 1416
3D7-DBL2X   668  TKDLELNLQKIFGKLFRKYIKKNNTAEQDTSYSSLDELRESWWNTNKKYIWLAMKHGAGMN 728
A4-DBL2X    670  TKDLELNLQNNFGKLFGKYIKKNNTAEQDTSYSSLDELRESWWNTNKKYIWTAMKHGAEMN 730
A4-DBL5ε    2118 FKDIKIKLDRLLEKET--N---------------NTKKAEDWWKTNKKSIWNAMLCGYKKS 2161
3D7-DBL5ε   2112 FKDIKRKLDRLLEKET--N---------------NTEKVDDWWETNKKSIWNAMLCGYKK- 2154
A4-DBL6ε    2447 IGKILGD--------TDGQ---------------N-EKRKKWWDMNKYHIWESMLCGYRE- 2482
3D7-DBL6ε   2444 IGKILGD--------GVGQN----------------EKRKKWWDMNKYHIWESMLCGYKHA 2485

                 HH---------------------------HHHHHHHHHHHHHHHHHHHHHHHHHHHHHHH--
A4-DBL3X    1423 NK---KNNNSIFN-GDECGVS--PPTGNDEDQSVSWFKEWGEQFCIERLRYEQNIREACTI 1478
3D7-DBL3X   1417 NKK--QKKNGTF-SIDECGIF--PPTGNDEDQSVSWFKEWSEQFCIERLQYEKNIRDACTN 1473
3D7-DBL2X   729  STTC-CGDGSVTGSGSSCDDI--PTI-DLIPQYLRFLQEWVEHFCKQRQEKVKPVIENC-- 783
A4-DBL2X    731  ITTC-NADGSVTGSGSSCDDI--PTI-DLIPQYLRFLQEWVENFCEQRQAKVKDVITNC-- 785
A4-DBL5ε    2162 --GNKIID-PSWCTI--------PTT-ETPPQFLRWIKEWGTNVCIQKQEHKEYVKSKCSN 2210
3D7-DBL5ε   2155 -SGNKIID-PSWCTI--------PTT-ETPPQFLRWIKEWGTNVCIQKEEHKEYVKSKCSN 2204
A4-DBL6ε    2483 -AEGDTET-NENCRFPD------IESV---PQFLRWFQEWSENFCDRRQKLYDKLNSECIS 2532
3D7-DBL6ε   2486 --YGNISE-NDRKMLD-------IPNNDDEHQFLRWFQEWTENFCTKRNELYENMVTACNS 2536

                 ---------------------HHHHHHHHHHHHHHHHHHHHHHHHHHHHHHHH---------
A4-DBL3X    1479 NGKNEKKCINSKSGQGDKI-QGACKRKCEKYKKYISEKKQEWDKQKTKYENK--------Y 1530
3D7-DBL3X   1474 N--------GQ--GDKI---QGDCKRKCEEYKKYISEKKQEWDKQKTKYENK--------Y 1513
3D7-DBL2X   784  -----KSCKES-GGTCNGECKTECKNKCEVYKKFIEDCKGGDGTAGSSWVKRWDQIYKRYS 838
A4-DBL2X    786  -----KSCKESGNKCKTEC-KTKCKDECEKYKKFIEACGTAGGGIGTAGSPWSKRWDQIYK 840
A4-DBL5ε    2211 VTNLGA---------QASE-SNNCTSEIKKYQEWSRKRSIRWETISKRYKKYKRMDILKDV 2261
3D7-DBL5ε   2205 VTNLGA---------QESE-SKNCTSEIKKYQEWSRKRSIQWEAISEGYKKY-------KG 2248
A4-DBL6ε    2533 AECTN--------G--SVD-NSKCTHACVNYKNYILTKKTEYEIQTNKYDNE--------F 2574
3D7-DBL6ε   2537 AKCNT---------SNGSVDKKECTEACKNYSNFILIKKKEYQSLNSQYDMN---YKETKA 2585

                 ------------------------------------------------------------HHHH
A4-DBL3X    1531 -----VGKS------------ASDLLKEN---YPECISAN-----FDFIFND-----NIEY 1561
3D7-DBL3X   1514 -----VGKS------------ASDLLKEN---YPECISAN-----FDFIFND-----NIEY 1543
3D7-DBL2X   839  KYIEDAKRNRKAGTKNCGPSSTTNAAENK------CVQSDIDSFFKH----LIDIGLTTPS 889
A4-DBL2X    841  RYSKHIEDAKR----------NRKAGTKN------CGTSSTTNAAASTDENKCVQ--SDID 883
A4-DBL5ε    2262 KEPD -----------ANTYLREH------CSK------------------------------ 2276
3D7-DBL5ε   2249 MDEF -----------KNTFKNIK---E----DAN--------EPNANEYLKKHCSK---- 2279
A4-DBL6ε    2575 KNKN SNDKD------APDYLKEK------CNDNKCEC--LNKHIDDKNKTWKNPYETLED 2620
3D7-DBL6ε   2586 EKKE -----------SPEYFKDKCNGECSCLSEY-----FKDETRW-----KNEVKNN-- 2622

                 HHH---HHH-------------------------------------------------
A4-DBL3X    1562 KTYYPYGDYSSICSCE----------------------------------------- 1577
3D7-DBL3X   1544 KTYYPYGDYSSICSCE----------------------------------------- 1559
3D7-DBL2X   890  -SYLSIVLDDNICGADK-APWTTYTTYTTTEKCN-------------- 921
A4-DBL2X    883  SFFKHLIDIGLTTPSSYLSNVLDDNICGADKAPWTTYTTYTTTEKCN 930
3D7-DBL5ε        ------------------------------------------------
A4-DBL5ε         ------------------------------------------------
A4-DBL6ε    2621 T--FKSKCDCPKP------------------------------------ 2631
3D7-DBL6ε   2623 -------CMCKPP------------------------------------ 2628
```
